# Single-nucleotide polymorphism rs1761667 in the *CD36* gene is associated with orosensory perception of a fatty acid in obese and normal-weight Moroccan subjects

**DOI:** 10.1017/jns.2020.18

**Published:** 2020-06-30

**Authors:** Habiba Bajit, O. Ait Si Mohammed, Y. Guennoun, S. Benaich, E. Bouaiti, H. Belghiti, M. Mrabet, E. M. Elfahime, N. E. El Haloui, N. Saeid, K. El Kari, A. Hichami, N. A. Khan, H. Benkirane, H. Aguenaou

**Affiliations:** 1 Ibn Tofaïl University – CNESTEN, Joint Research Unit in Nutrition and Food, Regional Designated Center of Nutrition (AFRA/IAEA), 14000 Kenitra, Morocco; 2 Research Team in Neurology and Neurogenetics, Genomics Center of Human Pathologies, Faculty of Medicine and Pharmacy, Mohammed 5th University, 10100 Rabat, Morocco; 3 Physiology and Physiopathology Research Team, Research Centre of Human Pathologies Genomics, Faculty of Sciences, Mohammed 5th University in Rabat, 4, Avenue Ibn Battouta BP 1014, Rabat, Morocco; 4 Laboratory of Epidemiology and Clinical Research, Faculty of Medicine, Mohammed 5th University, 10100 Rabat, Morocco; 5 Nutrition Unit Hygiene and Collectivity Medicine Ward, Military Hospital of Instruction Mohammed 5th, 10110 Rabat, Morocco; 6 HPC Ain Sbaa Groupe Akdital, Casablanca, Morocco; 7 Unités d'Appui Techniques à la Recherche Scientifique (UATRS), Plateforme Biologie Moléculaire et Génomique Fonctionnelle, Centre National pour la Recherche Scientifique et Technique (CNRST), 10102 Rabat, Morocco; 8 Physiologie de la Nutrition & Toxicologie, INSERM UMR 1231, Université de Bourgogne, Faculté des Sciences de la Vie, 21000 Dijon, France

**Keywords:** Obesity, BMI, Fat taste sensitivity, Oleic acid detection thresholds, *CD36*, SNP rs1761667, OA, oleic acid

## Abstract

Obese subjects have shown a preference for dietary lipids. A recent collection of evidence has proposed that a variant in the *CD36* gene plays a significant role in this pathway. We assessed the association between the orosensory detection of a long-chain fatty acid, i.e. oleic acid (OA), and genetic polymorphism of the lipid taste sensor CD36 in obese and normal-weight subjects. Adult participants were recruited in the fasting condition. They were invited to fat taste perception sessions, using emulsions containing OA and according to the three-alternative forced-choice (3-AFC) method. Genomic DNA was used to determine the polymorphism (SNP rs 1761667) of the *CD36* gene. Obese (*n* 50; BMI 34⋅97 (sd 4⋅02) kg/m^2^) exhibited a significantly higher oral detection threshold for OA (3⋅056 (sd 3⋅53) mmol/l) than did the normal-weight (*n* 50; BMI 22⋅16 (sd 1⋅81) kg/m^2^) participants (1⋅20 (sd 3⋅23) mmol/l; *P* = 0⋅007). There was a positive correlation between OA detection thresholds and BMI in all subjects; evenly with body fat percentage (BF%). AA genotype was more frequent in the obese group than normal-weight group. OA detection thresholds were much higher for AA and AG genotypes in obese subjects compared with normal-weight participants. Higher oral detection thresholds for fatty acid taste are related to BMI, BF% and not always to *CD36* genotype.

The epidemic of obesity is considered a serious issue and has become an alarming threat to human health in the world. Its prevalence is rising rapidly in all age groups. According to the WHO, worldwide obesity has more than doubled since 1980^(1)^; the Southern Mediterranean countries are also affected with this curse. According to the national survey, conducted by the High Commission for Planning (HCP; Haut-Commissariat au Plan) in 2011, there were 10⋅3 million overweight Moroccan adults and 3⋅6 million obese^(2)^.

There are several factors including genetics, environment and dietary habits that are involved in the incidence of obesity. Dietary fat is consumed in high amounts by obese subjects because of its olfactory, visual and textural cues^(3,4)^. Interestingly, recent evidence has supported the implication of gustatory and hedonic properties in high fat intake, and there might exist a new taste quality, i.e. ‘fat taste’^(5,6)^. Lingual CD36 (cluster of differentiation 36) has been shown to be implicated in this mechanism in taste bud cells^(7–9)^. It has been proposed that an alteration in the functionality of such a receptor might influence the individual's sensitivity, preference and detection of foods^(10)^. Recent studies have supported the existence of a high preference for dietary lipids in obese subjects compared with lean participants^(9–12)^. Hence, the lean and obese subjects have been termed, respectively, as ‘hypersensitive’ or ‘hyposensitive’ to fatty acids^(9,13)^. The decreased change in orosensory capacity has been further suggested to result in fatty food intake that will again aggravate the degree of obesity^(10,14,15)^.

The dysfunction of perception via CD36 could be influenced by a common SNP: rs1761667^(16)^. This SNP has been well studied in different populations, including Algeria, Tunisia and Italy, and has also been associated with the orogustatory perception of fatty acids. In particular, its alleles A and G have been linked, respectively, to a low and high sensitivity to detect fat taste^(8,9,16,17)^.

Keeping in mind the aforementioned observations on fat taste alteration in obesity and the implications of CD36, we investigated the role of the *CD36* rs1761667 SNP in the orogustatory perception of oleic acid (OA) in obese and normal-weight Moroccan subjects. This would be the first study on a Moroccan population.

## Methods

### Study design

#### Type, period and place

This is a non-interventional analytical case−control study, carried out for 1 year in collaboration with the Military Hospital of Instruction Mohammed 5th (M.H.I.M.5, Rabat, Morocco). The hospital is endowed with technology equipment, high quality of care and also the humanity of its staff which makes it the first medical reference structure for both Moroccan military and civilian patients.

### Population

#### Inclusion criteria

Participants (male and female, adults: age between 18 and 58 years old) were recruited from a group of patients visiting the Nutrition Unit of the M.H.I.M.5 for a general health check-up, on consultation days: Monday, Tuesday and Thursday. The subjects were divided into two groups: obese (BMI ≥30 kg/m^2^) and normal weight (BMI between 18⋅5 and 24⋅9 kg/m^2^) according to the WHO guidelines^(18)^.

#### Exclusion criteria

The exclusion criteria were as follows: subjects suffering from chronic illnesses (diabetes, hypertension, CVD, etc.), smokers, pregnant, lactating and/or menopausal women, patients undergoing treatment (known to affect taste quality), from the same family, mentally/physically handicapped people or subjects refusing to participate. Each patient recruited for the study was given clear information about its modalities and willingly agreed to participate.

### Ethics

The present study received the approval of the Ethical Committee and Biomedical Research (CEBR) of the Faculty of Medicine and Pharmacy, University Mohammed 5th in Rabat, Morocco (folder number: 38/15, delivered on 1 March 2018), in accordance with the ethical standards of the institutional and/or national research committee and as per the 1964 Helsinki Declaration and its later amendments or comparable ethical standards. Before undertaking the protocol, all objectives and modalities were clarified to the participants by an attending physician. Written and oral consent was therefore willingly obtained.

This trial was registered at http://www.pactr.org/ (Pan African Clinical Trials Registry) as PACTR201803003172179.

### Data collection

The collection was conducted by filling out a set of questionnaires including identity information, anthropometric measures and the results of oral fat detection thresholds and genetic polymorphism.

### Anthropometric measurements

All anthropometric parameters were determined with minimal clothing and without shoes. Body weight was measured to the nearest 0⋅1 kg using a scale (seca GmbH and Co. KG). Height was measured to the nearest at 0⋅1 cm using a stadiometer (seca GmbH and Co. KG). BMI was calculated as a ratio of weight in (kg) by the squared height in (m^2^). Waist circumference was measured using a steel measuring tape, with measurements made halfway between the lower border of the ribs and the iliac crest in a horizontal plane. Hip circumference was measured at the widest point over the buttocks. Additionally, body composition, in particular, body fat, was calculated with a simple instrument, an impedance meter (BodyStatQuadscan 4000).

### Biochemical analyses

Data on certain biochemical parameters including the concentrations of fasting glucose, insulin and lipid profile (total cholesterol, HDL-cholesterol, LDL-cholesterol and TAG) were obtained from the results of the biological sheet corresponding to each patient who was asked during his first consultation where he was recruited for this study. Analyses were performed by an external laboratory using routine standard techniques.

### Blood sample collection

Blood was collected from each participant for genotype analyses. The venous blood sampling on an EDTA tube of approximately 4 ml was performed by qualified personnel of the nutrition unit following good laboratory practices. Once collected, the tube was immediately capped, carefully stirred by inversion 5−10 times, then directly labelled and anonymised by codes specific to the study. All other information on the patient's identity, clinical diagnosis, storage code, etc. has been listed in a digital database (file on Epi Info software) and in another paper form (the patient observation book) to keep traceability. The samples obtained were stored at −80°C until genetic analysis.

### Oleic acid sensitivity test

OA sensitivity analysis was carried out using emulsions containing food-grade (Sigma) OA (a MUFA), in demineralised water, at ascending concentrations (0⋅018, 0⋅18, 0⋅37, 0⋅75, 1⋅5, 3, 6 and 12 mmol/l) according to the three-alternative forced-choice (3-AFC) method^(9,19)^. To prevent oxidation of OA, the solutions contained EDTA (Merck) at 0⋅01 % (w/v). The emulsions were homogeneously sonicated, keeping them cold, for 6−9 min (30 s of functioning and 60 s of stopping) in an ice bath. The application of sonication is used to make stable oil−water emulsions while guaranteeing sample homogeneity^(20)^. Acacia gum (Sigma) at 5 % (w/v) present in all the solutions served as a control to mimic the textural properties of the oils in the control solution^(20,21)^. The samples were prepared fresh on the day of testing in opaque polypropylene tubes (known to promote desorption of NEFA for the period of emulsion preparation, thus, limited the loss of glass surfaces)^(20)^ until being served at room temperature. In the same way, the control samples were prepared, but without adding fatty acid.

The participants were invited in groups of ten individuals on a specified day per week and suggested to come early in the morning in a fasting state, or at least not having eaten or drunk 2 h before the test. In an isolated chamber of the Hygiene Ward, the subjects were exposed to taste the three solutions one by one. One solution contained OA with acacia gum and the two others were served as controls with acacia gum only. We started with the lowest OA concentration. At that time, subjects were asked to rinse the mouth between every tasting and were not allowed to drink the solutions; rather, they had to spit them out after a brief passage in the mouth (gargling for a few seconds). They were asked whether they observed a difference in the taste sensation between the three samples; in the case of the failure in the fat taste sensation, the OA concentration was increased for the next set until they could detect its presence. The procedure continued until having identified three consecutive times the identified sample at the same OA concentration given, then the current value of concentration represented the detection threshold of the participant.

### SNP analyses

Genomic DNA (gDNA) was extracted from 200 μl of whole blood, using a simple and reliable protocol of purification from a commercial DNA isolation kit (ISOLATE II Genomic DNA Kit; Bioline), according to the manufacturer's protocol. The rs1761667 polymorphism of the *CD36* gene was genotyped using the PCR method. The gDNA was amplified with BIO-X-ACT™ Short Mix, containing BIO -X-ACT Short DNA Polymerase, MgCl_2_, ultra-pure dNTPs manufactured by Bioline as well as further additives (BIO-X-ACT™ Short Mix, Bioline) with forward (5′-CAA AAT CAC AAT CTA TTC AAG ACCA-3′) and reverse (5′-TTT TGG GAG AAA TTC TGA AGA G-3′) primers. The amplified DNA was sequenced with the BigDye^®^ Terminator v. 3.1 Cycle Sequencing Kit (Applied Biosystems), by using a ABI 3130xl Genetic Analyzer, 16 capillary sequencers (Applied Biosystems). The resulting sequences were analysed with sequencing analysis software, v. 5.3.1 (Applied Biosystems).

### Statistical analyses

Data entry was done by Epi Info software (version 3.5.4; Centers for Disease Control and Prevention) and statistical analyses were conducted by using SPSS software (Statistical Package for the Social Sciences, version 24; IBM). We tested the normality of each variable by the one-sample Kolmogorov−Smirnov test. The variables normally distributed were presented as mean values and standard deviations. The significance of measured parameters between the groups of our study was determined by Student's *t* test. The qualitative variables were expressed in percentages and were compared by the χ^2^ test. The Mann−Whitney test was used to compare the differences between variables in the studied groups. For correlation between BMI, body fat and OA detection thresholds, Spearman rank correlation was performed. Two-sided *P* values <0⋅05 were considered significant.

## Results

### Subject characteristics

The anthropometric parameters and clinical characteristics of participants are reported in Table 1. A total of 100 participants were recruited for the present study. They were divided into two groups based on their BMI: obese (*n* 50) with an average BMI of 34⋅97 (sd 4⋅02) kg/m^2^ and normal weight (*n* 50) with an average BMI of 22⋅16 (sd 1⋅81) kg/m^2^ (*P* = 0⋅000). The study included females (*n* 72) and males (*n* 28). Both groups had the same average age; the mean age was 32⋅37 (sd 9⋅52) years (*P* = 0⋅06). There was a significant difference in body fat percentage between obese (43⋅26 (sd 5⋅99) %) and normal-weight (25⋅66 (sd 4⋅68) %) subjects (*P* = 0⋅000). Also, a significant difference was observed in their waist and hip circumferences (*P* = 0⋅000).

**Table 1.** Clinical characteristics of obese and normal-weight groups (Mean values and standard deviations)

	Obese (*n* 50)		Normal weight (*n* 50)	
Parameters	Mean	sd	Mean	sd
Age (years)	35·30	9·25	39·44	8·94
Weight (kg)	91·56**	11·44	60·62	11·29
Height (cm)	162*	6·90	166	9·20
BMI (kg/m^2^)	34·97***	4·02	22·16	1·81
Waist (cm)	106·53***	9·11	84·69	8·55
Hip (cm)	119·24***	8·70	100·94	5·92
Body fat (%)	43·26***	5·99	25·66	4·68
FG (mmol/l)	6·55*	4·88	5·16	0·39
Insulin (pmol/l)	83·37*	18·73	59·27	16·65
TAG (mmol/l)	1·23*	0·35	0·91	0·24
TC (mmol/l)	4·71	0·77	4·66	1·39
HDL-C (mmol/l)	1·19	0·23	1·50	0·21
LDL-C (mmol/l)	3·00	0·60	2·74	1·37

FG, fasting glucose; TC, total cholesterol; HDL-C, HDL-cholesterol; LDL-C, LDL-cholesterol.

Mean values were significantly different from those of the normal-weight group: * *P* < 0·05, ** *P* < 0·01, *** *P* < 0·001 (Student's *t* test).

Obese participants had higher fasting glucose compared with the normal-weight group (*P* = 0⋅03). They had together insulin, TAG and total cholesterol concentrations within the normal range; nevertheless, the obese group had rather higher values than the normal-weight group, respectively (*P* = 0⋅02, *P* = 0⋅0422, *P* = 0⋅069). Similarly, HDL-cholesterol and LDL-cholesterol concentrations were normal in both groups; however, obese participants had lower HDL-cholesterol (*P* = 0⋅0710) and higher LDL-cholesterol than those of normal weight (*P* = 0⋅8409), but without a significant difference. No difference was observed between sexes regarding the above-mentioned parameters (Table 1).

### Orosensory detection of oleic acid

**Fig. 1.**
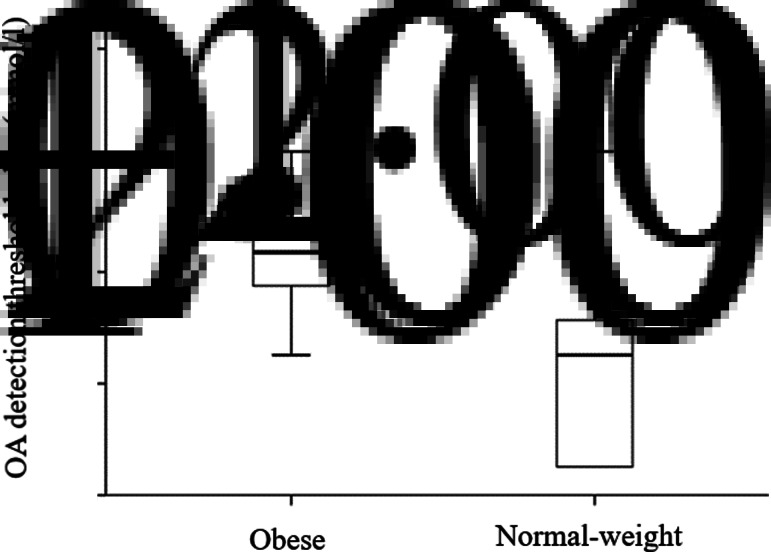
Oleic acid (OA) orosensory detection in obese and normal-weight subjects. The figure shows the box plots of medians, first and third quartiles, standard deviations, and extreme values for both obese (*n* 50) and normal-weight (*n* 50) groups. The average OA detection thresholds were greater in the obese than in the normal-weight group (*P* = 0⋅007; Mann−Whitney *U* test).

**Fig. 2.**
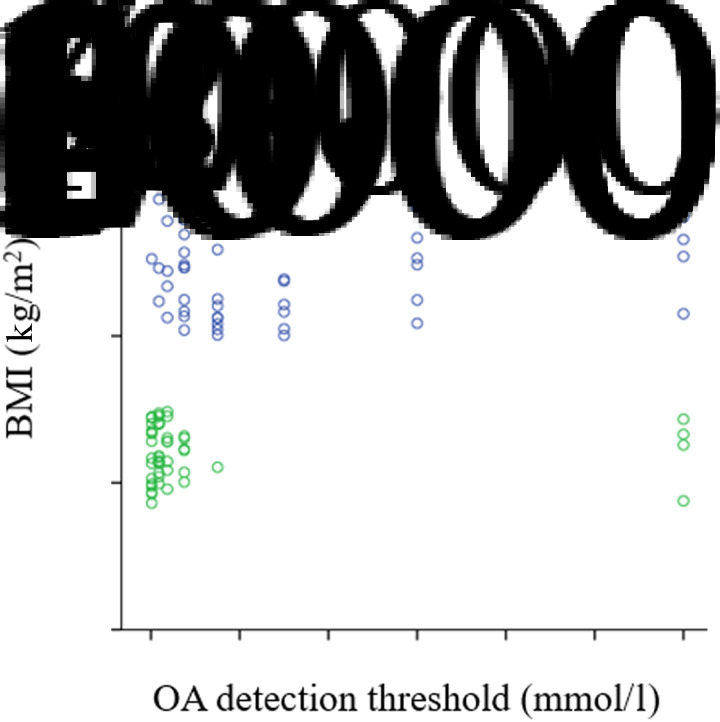
Relationship between BMI and oleic acid (OA) orosensory detection thresholds. Spearman rank correlation between BMI (kg/m^2^) and OA (mmol/l) orosensorial detection in all participants (*n* 100). A positive correlation was observed (*r* 0⋅274; *P* = 0⋅006). ο, Obese; ο, normal weight.

We noticed a statistically significant difference in OA oral detection thresholds between obese and normal-weight subjects. The average OA detection thresholds were greater in the obese group (3⋅056 (sd 3⋅53) mmol/l) than in the normal weight group (1⋅20 (sd 3⋅23) mmol/l; *P* = 0⋅007) (Fig. 1). Frequencies of the detection thresholds for OA by obese and normal-weight participants are reported in Table 2. There was a positive correlation between OA orosensory detection thresholds and BMI of our participants (*r* 0⋅274; *P* = 0⋅006) (Fig. 2).

**Table 2.** Oleic acid (OA) detection threshold frequencies in obese and normal-weight groups† (Numbers of subjects and percentages)

	Obese (*n* 50)		Normal weight (*n* 50)	
OA concentrations (mmol/l)	*n*	%	*n*	%
0⋅018	1	2	16	32*
0⋅18	3	6	15	30*
0⋅37	4	8	8	16*
0⋅75	13	26	6	12*
1⋅5	8	16	1	2*
3	9	18	0	0*
6	7	14	0	0*
12	5	10	4	8
Total	50	100	50	100

* Significant (*P* < 0⋅05) difference in the frequency of detection of OA compared with the obese subjects (χ^2^ test).

† Subjects were presented with each series of concentrations in ascending order, from the lowest (0⋅018 mmol/l) to the highest (12 mmol/l) concentration.

**Fig. 3.**
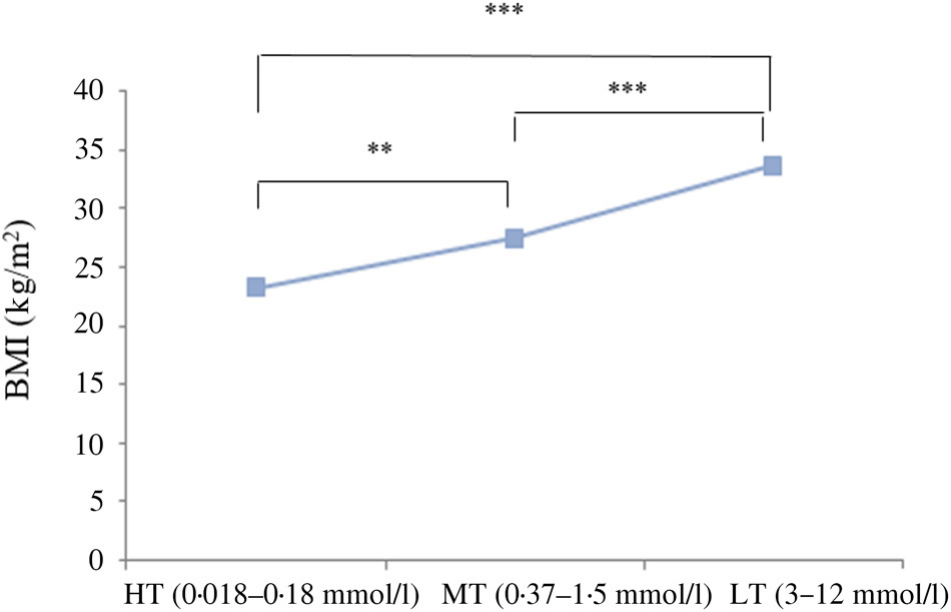
Oleic acid (OA) taste sensitivity in all subjects in relation to BMI. HT, high tasters (most are from normal-weight group, *n* 31 and 4 only from the obese group); MT, middle tasters (from both groups, obese: *n* 25 and normal weight: *n* 15); LT, low tasters (predominated by obese subjects, *n* 21 and just 4 are normal weight). The results are means. ** *P* < 0⋅01, *** *P* < 0⋅001 (Spearman's rank correlation).

**Fig. 4.**
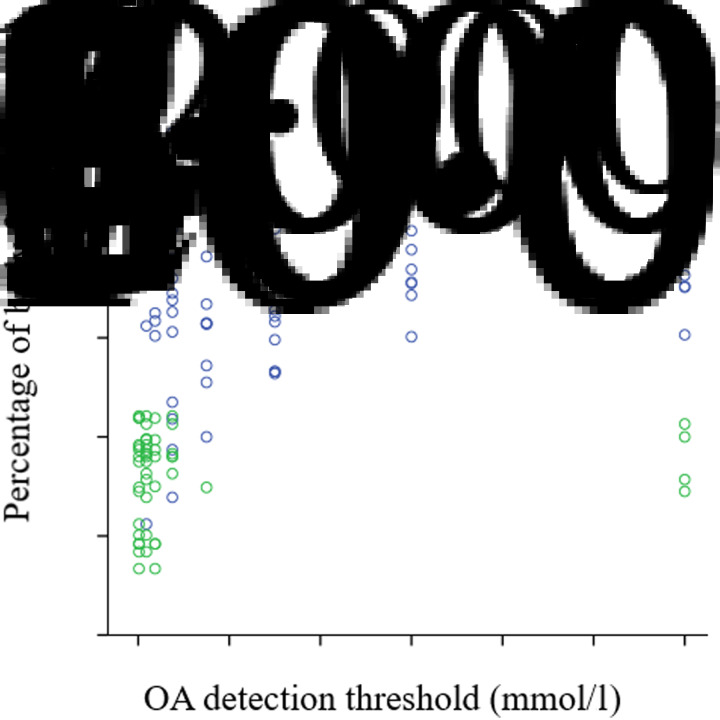
Relationship between fatty acid sensitivity and body fat distribution in obese and normal-weight subjects. The figure shows the Pearson correlation between body fat (%) and orosensory detection of oleic acid in all participants (*n* 100). A positive correlation was observed (*r* 0⋅330; *P* = 0⋅001). 
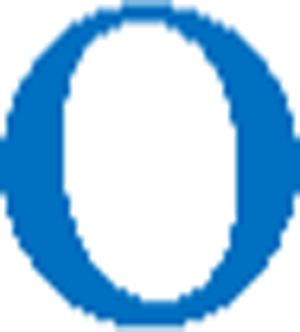
, Obese; 
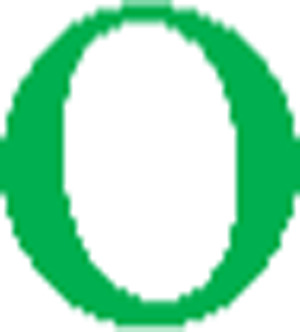
, normal weight.

Fig. 3 presents the fatty acid taste sensitivity for all the subjects in relation to their BMI. Depending on the concentrations of orosensory detection of OA, we could classify our participants into three categories: high tasters (between 0⋅018 and 0⋅18 mmol/l), middle tasters (between 0⋅37 and 1⋅5 mmol/l) and low tasters (between 3 and 12 mmol/l). Also, another positive correlation was observed between these OA detection thresholds and percentage of body fat distribution (*r* 0⋅330; *P* = 0⋅001) (Fig. 4). However, no significant difference was noticed between males and females in the measured parameters.

### SNP analyses

Genotype frequencies in *CD36* polymorphism are shown in Table 3. We compared the distribution of AA genotype with the other genotypes AG and GG. We observed a higher AA genotype frequency of rs1761667 in obese compared with normal-weight subjects (*P* = 0⋅049).

**Table 3.** Genotype distribution of *CD36* rs1761667 between obese and normal-weight subjects (Numbers of subjects and percentages)

	Obese (*n* 50)		Normal weight (*n* 50)		
Genotype	*n*	%	*n*	%	*P*
AA	16	32	9	18	0⋅049*
AG	26	52	32	64	0⋅224
GG	8	16	9	18	0⋅79

*CD36*, cluster of differentiation 36.

* There was a significant difference in the AA genotype frequency between obese and normal-weight subjects (χ^2^ test).

**Fig. 5.**
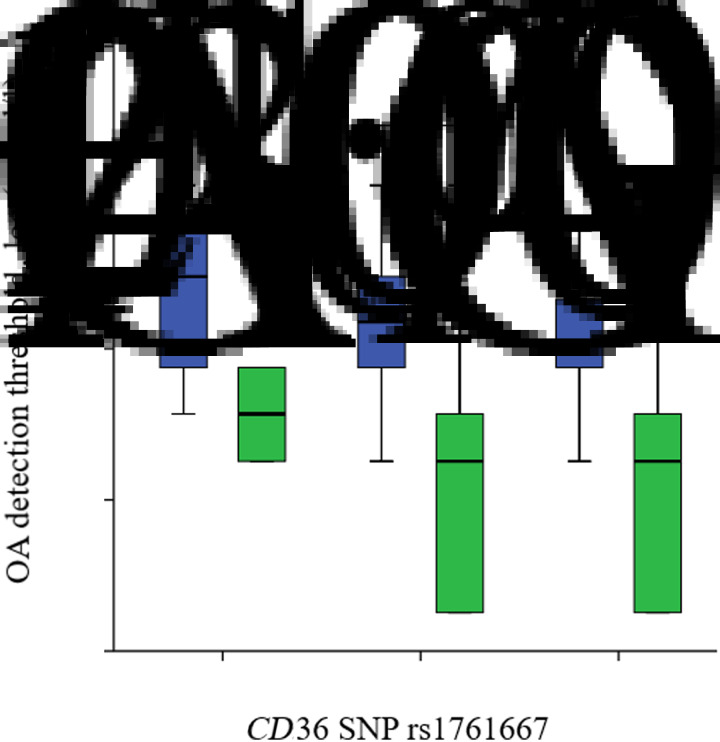
Relationship between oleic acid (OA) detection thresholds and genetic polymorphism of the cluster of differentiation 36 (*CD36*) in obese and normal-weight subjects (after cleaning data from outliers). Our participants (*n* 100) had either the AA (*n* 25), AG (*n* 58) or GG (*n* 17) genotypes of the *CD36* gene. The figure shows the box plots of medians, first and third quartiles, standard deviations and extreme values for both obese (*n* 50) and normal-weight groups (*n* 50). The genotype analyses of *CD36* rs1761667 were performed with the Mann−Whitney *U* test. Values were significantly different from those of the normal-weight group for AA and AG genotypes: * *P* < 0⋅05, ** *P* < 0⋅01. 
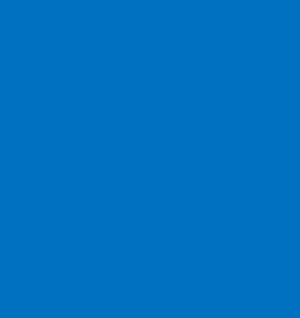
, Obese; 
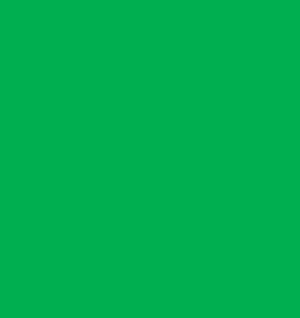
, normal weight.

Obese participants of the two genotypes AA (*P* = 0⋅004) and AG (*P* = 0⋅032) of SNP rs1761667 had higher detection thresholds with significant difference than those of normal weight (Table 4, Fig. 5), except for the GG genotype, for which the second group exhibited higher detection, but without significant difference (*P* = 0⋅606). After comparing the OA detection threshold in different genotype groups (comparing AA/AG and AA/GG, respectively), we observed that no significant difference was noticed among the three genotypes whether it was an obese (*P* = 0⋅224 and *P* = 0⋅076) or a normal-weight (*P* = 0⋅596 and *P* = 0⋅196) group.

**Table 4.** Oleic acid detection thresholds (mmol/l) and genotype distribution between obese and normal-weight subjects (Mean values and standard deviations)

	Obese (*n* 50)		Normal weight (*n* 50)		
Genotype	Mean	sd	Mean	sd	*P*
AA	4⋅20	4⋅38	0⋅44	0⋅32	0⋅004**
AG	2⋅74	3⋅24	0⋅96	2⋅91	0⋅032*
GG	1⋅80	1⋅90	2⋅79	5⋅22	0⋅620

Mean values were significantly different between obese and normal-weight subjects for those with AA and AG genotypes: * *P* < 0⋅05, ** *P* < 0⋅01 (Mann−Whitney *U* test).

## Discussion

Emerging evidence from several large studies have suggested the existence of fat taste as a sixth taste modality, which is dedicated to the orosensory perception of dietary lipids^(5,6,8,22)^. Compelling reports have shown that the lipid receptor CD36, mostly expressed in human circumvallate papillae^(5)^, seems to play a significant role in lipid detection. In the present study, we investigated the sensitivity to oral fatty acid detection (OA as a reference product) and the implication of *CD36* rs1761667 SNP in obese and normal-weight Moroccan adult subjects.

Concerning the biochemical profile of our participants, the obese group had normal fasting glucose levels compared with the normal-weight group. Both obese and normal-weight subjects exhibited normal concentrations of insulin, TAG and total cholesterol, except that the obese group had higher values than the normal-weight group. Also, the obese group showed slightly higher TAG concentrations. HDL-cholesterol and LDL-cholesterol values were normal in both groups, but without a significant difference between obese participants and those of normal weight. However, no difference was observed between males and females regarding all these parameters.

The major findings of OA detection thresholds were as follows: obese and normal-weight participants detected this fatty acid in their oral cavity at different concentrations. There was a large distribution of oral detection thresholds of OA in both groups, which reached the four orders of classification. This might have been attributable to the lack of experience or non-familiarisation with this type of test or stimulus. There was a significant difference in sensitivity and detection thresholds for OA between obese and normal-weight subjects. We noticed that oral sensitivity to fatty acid detection thresholds was related not only to the BMI of the participants, but also to their body fat percentage.

The present data confirm that obese subjects possessed a significantly higher threshold (lower sensitivity) for oral fatty acid detection compared with normal-weight subjects. These results are compatible with previous studies performed on Australian and Algerian teenagers and Tunisian adults^(7,8,13)^. Also, the data presented here suggest the possibility that individuals could be classified into hyposensitive (higher thresholds) or hypersensitive (lower thresholds) to dietary lipids, based on their fat taste detection thresholds as already demonstrated by previous experiments^(10,23)^. While ‘high tasters’ were mostly from the normal-weight group, ‘low tasters’ were predominately obese subjects. However, we observed the existence of another kind of participants, ‘non-tasters’, with a number of eight, where they failed to detect the fatty acid at any of the concentrations. They were all obese and were excluded from the present study. These results corroborate other reports mentioned in this article^(7,8,24)^. Similarly, we noticed another point where the oral sensitivity to OA was related to the BMI of participants. Subjects with greater BMI values (hyposensitive) had higher detection thresholds for OA, whereas those with low BMI values (hypersensitive) had lower thresholds, as demonstrated elsewhere^(7,13)^. In our study, we took into account the body fat percentage, which reflected exactly the adiposity of our participants. Our finding has revealed a positive link between oral fat taste sensitivity and body fat, which is very interesting because no such study has established this association before.

All these observations suggest that high detection thresholds or low sensitivity for fat taste may play a contributing role in the development of obesity. So, it is worthy to shed light on the implication of genetic factors in this situation^(25)^. *CD36* SNP is a candidate of fat taste marker. Hence, we observed that the obese group had a slight AA genotype frequency of *CD36* SNP rs1761667 compared with the normal-weight group. Also, we noticed slightly higher A-allele frequency compared with G-allele in the obese group; the same distribution has been reflected in some other Maghreb countries like Algeria^(7,9)^ and Tunisia^(9,26)^. This is very interesting, the fact that A-allele frequency was associated with reduced CD36 expression and higher detection thresholds for OA^(16)^. Therefore, these subjects might present an altered perception of fat taste and then consume more dietary lipids in their daily foods. Unlike other studies from different populations, including India^(27)^, Asia^(28)^ and Italy^(17)^, they indicated a high frequency of G-allele and not A-allele.

Our results also showed a relationship between *CD36* SNP and high OA detection thresholds in obese compared with normal-weight participants. In the present study, after comparing OA detection thresholds in the three genotype groups, we observed that the obese group with AA and AG genotypes for *CD36* SNP (rs1761667) possessed higher detection thresholds for fat taste than did subjects with normal weight. However, the concentrations obtained from obese individuals with the AA genotype are dispersed; this can have several explanations among which is the sample size of participants with this genotype is too small (<20). The other reason to explain this dispersion is habitual food consumption. Although we considered several parameters to include our subjects, dieting and recent weight loss were excluded criteria. This raises a question about the fatty food intake of our participants and its association with their detection thresholds. For example, a very recent study has reported that regular fatty food intake may reduce the individual's orosensorial response to fat^(29)^. From our data, we could not presume this fact in our participants since we had not studied their dietary intake, but we suppose the existence of an association between a high-fat diet and increased OA detection thresholds as our results showed a link between body composition and OA acid taste sensitivity; as already mentioned by other studies^(10,11)^.

Surprisingly, while trying to find an influence of *CD36* genotype on low OA sensitivity (high detection thresholds) in obese subjects as already noticed in other studies on obese women^(8)^ and lean and obese children^(9)^, we failed to find such an association in each group separately. Thus, no significant difference was observed between different genotypes even if it was an obese or a normal-weight group. The origin of this contradiction is not understood, but we can explain it in the case of Algerian children with the fact that maybe their papillae were less developed to express sufficiently the CD36 protein^(30)^. Actually, it has been demonstrated that the posterior region of the tongue rich in circumvallate papillae keeps on growing till the age of 15−16 years, contrary to the anterior region full of fungiform papillae, which reaches its adult size by the age of 8−10 years^(31)^. On the other hand, maybe that other variant of *CD36*^(26,32)^ or even of other genes like *GPR120* (another lipid receptor)^(33,34)^, might be possibly implicated in the alteration of orosensory detection of fatty acids in obesity. Despite all these dissimilarities, our finding is compatible with two studies that dealt with the same situation, for example those from Tunisian adults^(26)^ and from Algerian teenagers^(7)^, in which it was a case−control study comparing obese with normal-weight subjects. From the first study it was shown that the fatty acid detection threshold was not high for AA SNP (rs1761667) in the obese group which seems similar to our finding. As for the second study, it was mentioned that no difference exists between this genotype that was also presented predominately in the obese participants and OA oral sensitivity detection threshold in this group of teenagers.

Otherwise, there exists another feature which is the humoral aspect that may also account for our results, even though the dosage of adipokines was not the subject of this study. It is well known that all through obesity, changes in adipokine (adiponectin, leptin, etc.) levels may occur. For example, it has been shown that obesity decreases the proportion of adiponectin in humans^(35,36)^. Though, it was reported that this adipokine reduces hepatic CD36 expression levels^(37)^ which might disturb its functionality, since we have already mentioned the importance of CD36 as a fatty acid receptor and then impairs the fat taste detection. Likewise, from a recent study another adipokine, ‘leptin’, whose levels are much higher during obesity was reported to diminish the taste and olfactory capacity^(38)^. Therefore, these metabolic complications resulting from obesity may play a role in the alteration of orosensory taste perception of our participants.

### Conclusion

Certainly, our data are in confirmation with earlier observations and our experimental protocol conforms with the relevant human research ethical guidelines; however, we failed to show an association of high oral detection thresholds for fat taste and AA genotype of the *CD36* gene in obese subjects, though they exhibited higher levels of the *CD36* A allele than G allele. So, we cannot rule out the limitation of the present study and the recruitment place that may not reflect the general community. Besides, there is a need for further studies in other populations with different genotypes, culture and different eating habits in order to collect more evidence and information about the fat-sensing mechanisms involved in obesity. To conclude, our study is the first in Morocco to elucidate the relationship between fat taste sensitivity, genetic polymorphism and obesity. We have mentioned that obese participants exhibit a higher perception for fat than do normal-weight subjects; also, even though the obese group had a *CD36* AA genotype, it was not related to a high orosensory detection for OA. Therefore, our data are not sufficient to state if an altered oral fat perception affects BMI or leads to dietary fat over-consumption, and then promoting obesity in adult subjects. We suggest studying the cellular (taste bud cells) and metabolic mechanisms along with the endocrine factors (adipokines) involved during obesity on a large number of participants in the future. Also, complementary investigations (physiological tests) are needed, as modifications in the composition of saliva^(39)^, microbiota^(40)^ and/or in oral inflammation^(41)^ that affect the chemoreception of fatty acids are alternative hypotheses.
